# Radiation exposure and safety in low-dose CT-guided glycerol rhizotomy for trigeminal Neuralgia outside the operating room

**DOI:** 10.1007/s00701-024-06364-9

**Published:** 2024-11-22

**Authors:** Jiri Dostal, Jan Baxa, Jana Stepankova, Miroslav Seidl, Jan Mracek, Pavel Lavicka, Tomas Malkus, Vladimir Priban

**Affiliations:** 1 Department of Neurosurgery, Faculty of Medicine in Pilsen, Charles University, University Hospital, Pilsen, Czech Republic; 2 Department of Imaging Methods, Faculty of Medicine in Pilsen, Charles University, University Hospital, Pilsen, Czech Republic; 3 Department of Radiological Physics, Faculty of Medicine in Pilsen, Charles University, University Hospital, Pilsen, Czech Republic; 4 Department of Neurosurgery, Faculty of Medicine, University of Ostrava, University Hospital, Ostrava, Czech Republic

## Abstract

**Background:**

Percutaneous rhizotomy of the Gasserian ganglion is a well-established intervention for patients suffering from refractory trigeminal pain, not amenable to pharmacological management or microvascular decompression. Traditionally conducted under fluoroscopic guidance using Hartel’s technique, this study investigates a modified approach employing low-dose CT guidance to achieve maximal procedural precision and safety with the emphasis on minimizing radiation exposure.

**Methods:**

A retrospective analysis of patients undergoing percutaneous rhizotomy of the Gasserian ganglion at our institution was undertaken. Procedures were divided into fluoroscopy and CT-guided foramen ovale (FO) cannulation cohorts. Radiation doses were assessed, excluding cases with incomplete data. The study included 32 procedures in the fluoroscopy group and 30 in the CT group.

**Results:**

In the CT-guided group, the median effective dose was 0.21 mSv. The median number of CT scans per procedure was 4.5, and the median procedure time was 15 min. Successful FO cannulation was achieved in all 30 procedures (100%). In the fluoroscopy group, the median effective dose was 0.022 mSv, and the median procedure time was 15 min. Cannulation of FO was successful in 31 of 32 procedures (96.9%).

The only complications in the CT-guided group were three minor cheek hematomas. Immediate pain relief in the CT-guided group was reported in 25 of 30 procedures (83.3%), 22 of 30 (73.3%) provided relief at one month, and 10 of 18 (55.6%) procedures resulting in pain relief at one month continued to provide relief after two years.

**Conclusion:**

Low-dose CT-guided percutaneous rhizotomy conducted in the radiology suite carries negligible radiation exposure for patients and eliminates it for personnel. This method is fast, simple, precise, and carries a very low risk of complications.

## Background

Percutaneous rhizotomy of the Gasserian ganglion is a well-established procedure suitable for patients experiencing intractable trigeminal pain, particularly those unresponsive to pharmacological therapy and ineligible for microvascular decompression. Traditionally, the cannulation of the foramen ovale (FO) has been performed under fluoroscopy guidance using C-arm, following Hartel’s technique with surface landmarks of the patient’s head [7, 10]. This method, being only an estimated trajectory of the needle, can sometimes necessitate repeated punctures to cannulate the foramen ovale or result in a complete failure of cannulation. Additionally, repeated punctures carry a risk of facial pain, hematoma or infection [9, 15]. Therefore, various alternative methods to improve the needle placement control, such as neuronavigation or intraoperative CT scans using a standard or surgical cone-beam CT scanner, have been described [2, 4, 6, 17, 19, 21].

However, especially in cases requiring multiple procedures over a patient’s lifetime, the use of imaging methods can expose patients and personnel to significant radiation doses. This cumulative radiation exposure raises concerns about potential stochastic effects [5, 12]. While the safety and efficacy of various techniques of foramen ovale cannulation are well-documented, the data regarding specific radiation doses patients receive during these procedures are lacking, particularly when comparing fluoroscopy to modern imaging methods like low-dose CT.

At our institution, we have implemented a modification to the procedure by replacing fluoroscopy guidance with low-dose CT control for the cannulation of the foramen ovale, aiming to enhance both the precision and safety of the procedure. This modified approach is conducted using a standard CT scanner in our radiology department, outside of the operating room (OR) setting. Our procedure also excludes any radiation dose for the personnel. This study aims to provide a comparative analysis of the radiation doses associated with low-dose CT-guided FO punctures versus standard fluoroscopy-guided procedures. Additionally, it aims to establish the safety and efficacy of this modified approach.

## Methods

A retrospective review of percutaneous rhizotomies of the Gasserian ganglion conducted at our department was undertaken. Patients were categorized into two groups: those who underwent fluoroscopy-guided cannulation of the foramen ovale (FO) and those who underwent CT-guided cannulation of the FO. Fluoroscopy-guided procedures included in this study were conducted between 2008 and 2019 and 32 procedures performed on 24 patients were included. 18 patients underwent a single procedure, 5 patients underwent 2 procedures and one patient underwent 4 procedures. Fluoroscopy-guided procedures were replaced by CT-guided procedures in 2019, and they have been performed up to the present (2024). 30 procedures performed on 23 patients were included. 17 patients underwent a single procedure, 5 patients underwent 2 procedures and one patient underwent 3 procedures.

Three patients were excluded from the fluoroscopy-guided group because the data regarding radiation dose were insufficient to calculate the effective dose. No patients were excluded from the CT-guided group.

The patients included in the study ranged from 32 to 78 years of age. Fluoroscopy-guided procedures were conducted by a single senior neurosurgeon with more than 30 years of experience. CT-guided procedures were conducted by two neurosurgeons with 8 and 11 years of experience, respectively.

For the comparison of radiation exposure, the effective dose was chosen as it is a parameter calculable for both CT and fluoroscopy. The calculation was based on parameters obtained from radiation dose reports for each exposure. To calculate the effective dose from CT, ImpactDose 2.3 *(CT Imaging GmbH*,* Germany)* was used, utilizing parameters from patients’ CT radiation dose reports, including the volumetric CT dose index (CTDIvol, mGy), dose-length product (DLP, mGy × cm), applied voltage (kV), used mA, used filtration, rotation time (s), and pitch factor. For calculating the effective dose from fluoroscopy, PCXMC 2.0 *(STUK*,* Finland)* was used. The estimate was determined using parameters such as the displayed Dose-Area Product (mGy × cm²), applied voltage (kV), used filtration, radiation field size (cm), projection type, and patients’ BMI.

Procedures performed before 2008 were excluded from the analysis due to unavailability of reporting caused by a transition in the clinical IT system of our institution. Patients with incomplete radiation dose reports were also excluded from the study.

### Procedure

**Fig. 1:**
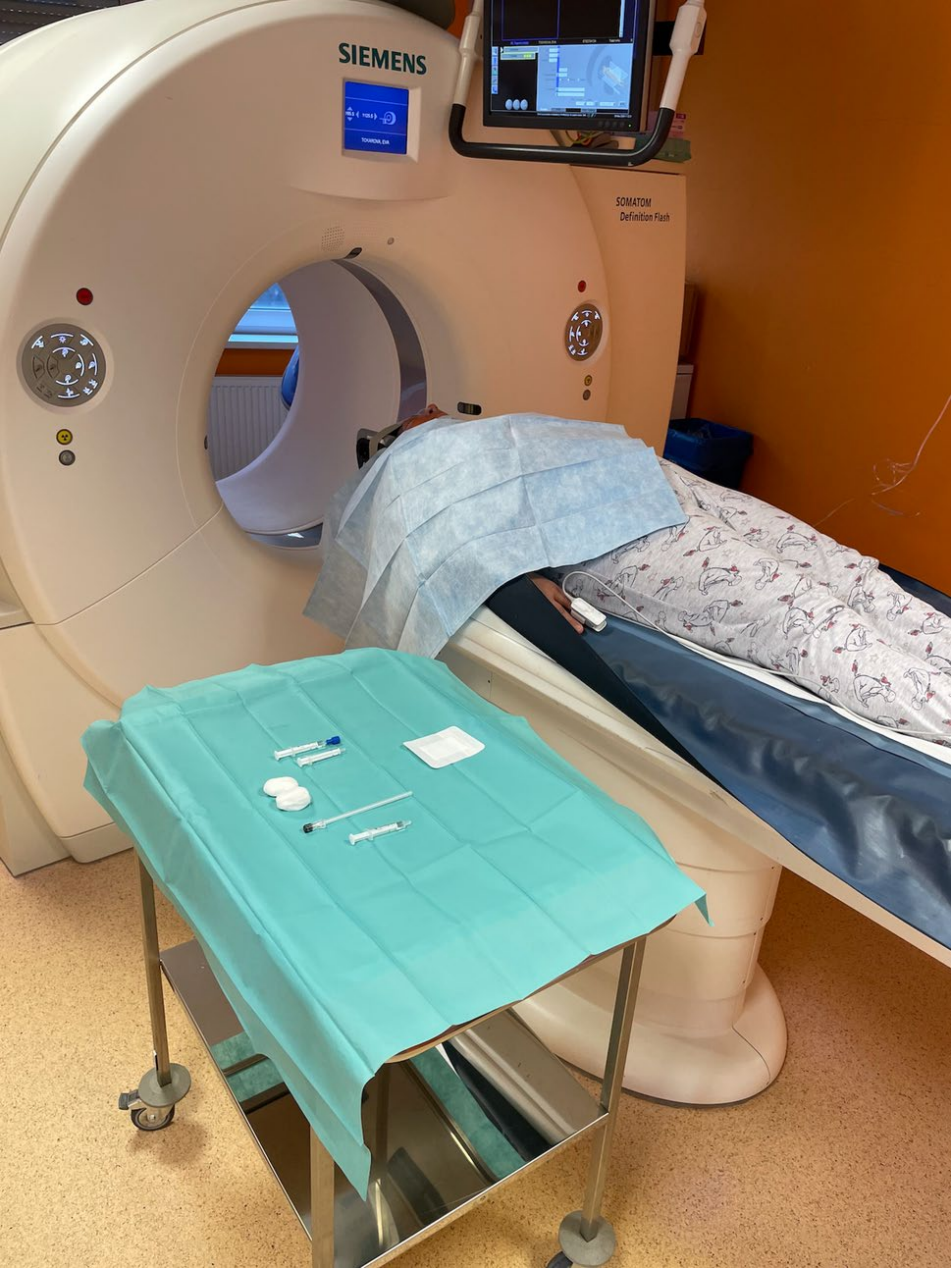
Arrangement of the room and patient during the procedure

The CT-guided procedures are conducted at the radiological department under the assistance of personnel experienced in percutaneous procedures. The patient is positioned in a supine position with the head secured within a head cradle on the CT bed. Anatomical landmarks, as per Hartel’s technique, are identified on the skin, and an entry point is marked [8]. The patient receives analgosedation, and vital signs are continuously monitored (Fig. 1). The procedure is not conducted under antibiotic prophylaxis.

**Fig. 2:**
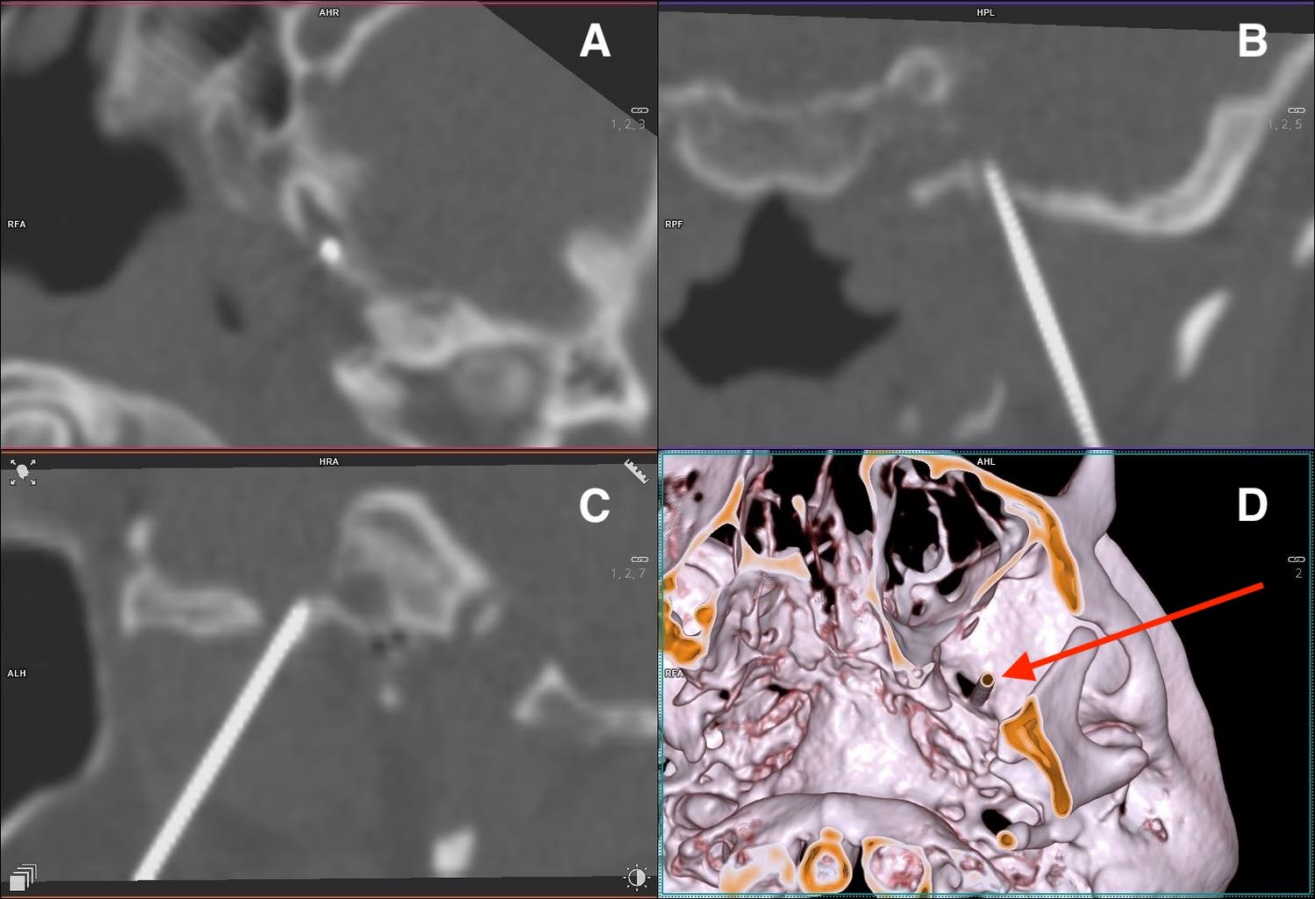
Position of the needle in the left foramen ovale. Projections: **A** – axial, **B** – coronal, **C** – sagittal, **D** – 3D reconstruction

A 22Gx90 mm needle is guided in a standard manner until it encounters bone, eliciting a jaw jerk or painful reaction, or reaches a depth of 8 cm. Subsequently, the personnel vacate the room, and a CT scan capturing only a necessary region of the skull base is conducted (Somatom Definition Flash CT Scanner, manufactured by Siemens Healthineers, Germany). CT scanning energy is lowered to 70 kV. Reconstructions of the CT images both in 2D and 3D are generated by an experienced radiologist, allowing visualization of the needle in relation to the skull base and the foramen ovale (Fig. 2). The needle position is adjusted based on CT scan findings, with repeat scans performed until the surgeon suspects the needle has entered Meckel’s cave, followed by a final scan for confirmation.

**Fig. 3:**
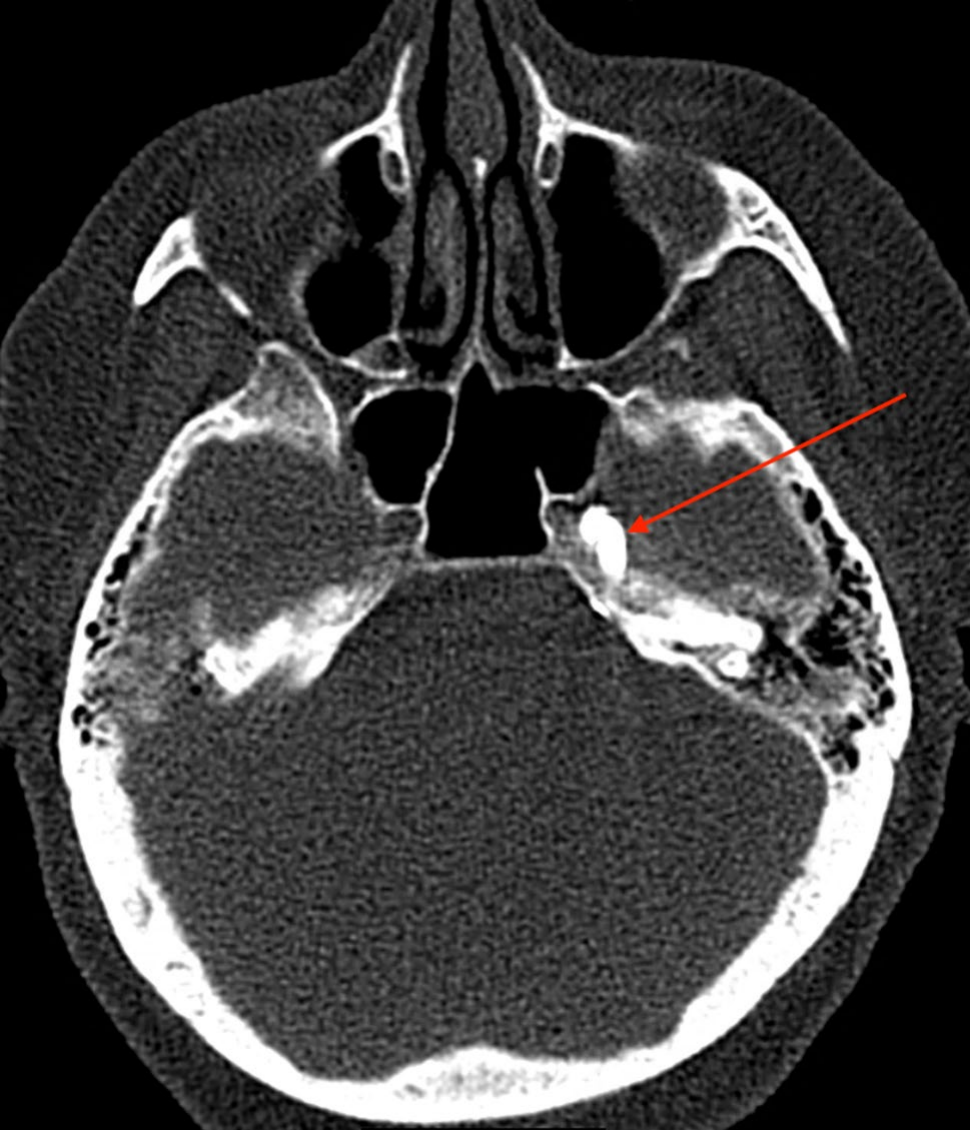
Red arrow showing contrast agent filling left-sided Meckel’s cave on axial CT scan

If the CT scan confirms the needle tip is in Meckel’s cave, a 2 ml syringe is used to aspirate cerebrospinal fluid (CSF). If blood is aspirated, the needle is readjusted. If CSF or no fluid is aspirated, 0.2 ml of iodine contrast agent is injected (Iomeron 300 mg/ml, manufactured by Bracco, Italy), and another CT scan is performed to assess the filling of Meckel’s cave with the contrast agent (Fig. 3). If satisfactory filling is observed, the patient is positioned in a sitting position with 30° head flexion [16]. Then, 0.4–0.6 ml of 85% glycerol is slowly injected in small increments. Positioning of the patient into a sitting position occurs on the CT bed with the assistance of the nurse and anesthesiologist. After the administration of glycerol and the removal of the needle, the patient is transferred to a ward bed. The patient remains in a sitting position, holding a large pillow against the abdomen and leaning forward, while the head of the bed is elevated to provide support to the patient’s back. The patient is then transferred from the CT suite to the ward and placed in an intermediary room for at least four hours.

After the procedure, the patient remains in the same position for at least 2 hours, during which vital functions and clinical state are closely monitored. The patient is discharged the following morning. A follow-up is scheduled for one month later. The patient should report any recurrence of pain or complications. If the result is satisfactory, the patient is referred back to their neurologist for further care. If the rhizotomy fails, other options, such as repeated rhizotomy or gamma knife irradiation of the root entry zone of the trigeminal nerve into the brainstem, are discussed.

Operating times are recorded by the circulating nurse. The beginning of the surgery is marked by the introduction of the needle into the cheek, and the surgery ends upon completion of the glycerol injection and the transfer of the patient to the ward bed.

The fluoroscopy guided procedures were conducted in the operating room. The patient was positioned supine, and the same entry point and landmarks as in CT guided group were used to achieve cannulation of the foramen ovale. The puncture trajectory and the location of the needle inside Meckel’s cave and its filling with the contrast agent were controlled using C-arm fluoroscopy. The same amount of contrast agent and glycerol was used. After the procedure, the patient’s head was positioned in anteflexion, similar to our CT-guided group.

### Analgosedation protocol

Analgosedation is performed by the same anesthesiologist from our team throughout every procedure. The patient is admitted one day prior to the procedure and is examined by the anesthesiologist on the day of admission. Chronic medications are not discontinued unless they interfere with the sedation protocol.

On the day of the procedure, no premedication is used. Two peripheral intravenous lines (20G, pink) are inserted. The patient is transferred to the CT suite and positioned on the CT scanner bed. Heart rate, oxygen saturation, and respiratory rate are monitored continuously throughout the procedure. Oxygen is administered at a flow rate of 4 L/min via a nasal cannula, allowing access for the puncture. The patient maintains spontaneous ventilation throughout the procedure.

Five minutes before the procedure begins, the patient is intravenously administered midazolam at a dose of 0.05–0.1 mg/kg and 5 µg of sufentanil. During the procedure, an additional 2.5 µg of sufentanil is administered after the third attempt to puncture the foramen ovale and prior to the glycerol injection. If the procedure lasts more than 20 min, an additional 2.5 mg of midazolam is given. No other analgesics are administered during the procedure. In case of a vagal reaction, 0.25–0.5 mg of atropine is administered intravenously, depending on the severity.

After the procedure, the patient is monitored in an intermediate care unit for 4 h, with the same parameters as during the procedure. Analgesics are administered on an individual basis; if necessary, we routinely use 20 mg of parecoxib or 1000 mg of metamizole intravenously. Higher doses of analgesics are provided for patients with a history of opioid use.

### Follow-up

The standard follow-up after the procedure at our department is either via telephone or an in-person visit to the office. The first contact occurs at two weeks to one month after the procedure. Additional contact can be made at any time in case of any issues or pain recurrence. This follow-up regimen was historically applied for fluoroscopy-guided procedures and has not been changed for CT-guided procedures.

The reported two-year follow-up is not part of the standard procedure but was conducted via telephone for the purpose of this study. All patients from the CT group were contacted and asked a series of simple questions regarding the effect of the procedure and complications by the neurosurgeon and the head nurse of the Department of Neurosurgery, both of whom are part of the team that performed the procedure (Table 1). All patients responded to the phone call, answered all the questions, and stated that they either remembered or were able to find precise information regarding the questions asked. The obtained data were transferred to the Barrow Neurological Institute (BNI) pain intensity score (Table 2) [3], and any complications were noted. Pain relief was classified using the BNI score, as grades 1 to 3. BNI grades 4 and 5 were classified as unsatisfactory results. Patients who no longer experience relief from the pain were offered a follow-up consultation at our outpatient clinic.

**Table 1:** A series of questions asked to patients during a phone follow-up

Do you still feel relief from the pain? Is it complete?
If not, how long did the relief from the pain last?
Do you use any analgesics on a daily basis?
Do you or did you have any sensory disturbances in your face on the side of the procedure?
Do you or did you have any problems with your eyes?
Did you have face hematoma?
Do you or did you feel any mastication weakness?
Did you experience any problems immediately after the procedure?
Do you or did you have any other issues or concerns?
Do you visit a neurologist regularly?

**Table 2:** Barrow Neurological Institute (BNI) pain intensity score

Score	Pain description
1	No pain, no medications
2	Occasional pain, no medications required
3	Some pain, adequately controlled with medications
4	Some pain, not adequately controlled with medications
5	Severe pain or no pain relief

## Results

In the CT-guided group, median effective dose was 0.21 mSv (range 0.11–1.02 mSv), median number of CT scans in a single procedure was 4.5 (range 3–17). Median time of procedure was 15 min (range 10–35 min). Cannulation of FO was successful in all 30 cases (100%).

In the fluoroscopy-guided group, median effective dose was 0.022 mSv (range 0.001–0.191 mSv), median time of procedure was 15 min (range 10–38 min). Cannulation of FO was successful in 31 of 32 cases (96.9%).

In the CT-guided group, 25 of 30 procedures (83.3%) resulted in immediate relief from pain, with 22 of 30 (73.3%) providing relief at the one-month follow-up appointment. Among the 20 procedures in patients who already completed the two-year follow-up, 10 of 18 (55.6%) procedures resulting in pain relief at one month continued to provide relief after two years.

In the CT-guided group, there were three mild cheek hematomas that resolved within one month. We observed no other complications, including corneal hypesthesia, more than mild facial sensibility loss, dysesthesia, or meningitis.

## Discussion

The method we present could potentially offer several advantages over conventional cannulation techniques. Firstly, this approach achieved successful cannulation of Meckel’s cave in 100% of patients, with no serious complications observed. It also involved a negligible radiation dose for patients and completely eliminated radiation exposure for medical personnel. The method is easily reproducible, requiring only a standard CT scanner and offering a steep learning curve. Patients tolerated analgosedation well, with no need for airway management. The pain relief observed in our study was consistent with previously published results. Given the procedure’s safety, simplicity, and patient tolerability, it can be safely repeated without concerns about cumulative radiation exposure.

Precise puncture of the foramen ovale can be challenging in some patients. While specific landmarks and approaches have been extensively described [8, 15, 22], they do not account for individual variations in skull base anatomy. Some studies reported a failure rate of cannulation of Meckel’s cave of up to 16% [11] and percutaneous puncture of the foramen ovale can also be associated with rare but severe complications, particularly along the depth of the puncture trajectory. The risks include injury to the internal carotid artery and cranial nerves, as well as the occurrence of intracranial hematomas [9]. They can be minimized through improved radiographic visualization techniques [13, 15]. Several advanced approaches were published. Flat panel or cone beam CT have been employed to confirm or guide needle placement in the foramen ovale [1, 4, 19]. Bohnstedt et al. successfully utilized intraoperative navigation with cone-beam CT to access the FO in challenging cases [2]. While the main advantage of controlling the entire puncture trajectory is evident, there are some drawbacks to the described procedures. Cone beam CT or similar devices may not be readily available to all providers, or it may be extensively utilized for spinal surgery or angiography, limiting the availability of time for FO cannulation procedures. Schmidt et al. reported using a personalized approach with an initial CT scan to mark the entry point based on the individual anatomy of the skull base and the position of the foramen ovale [17].

The use of glycerol offers several advantages, notably resulting in milder and fewer complications compared to radiofrequency ablation or balloon compression [11]. Another advantage is the simplicity and short duration of the procedure. It also eliminates the intraoperative need for sensory testing and carries a lower risk of facial sensory loss compared to other methods. However, its primary drawback is the variability in effectiveness, with reported pain relief outcomes ranging widely [10, 16, 18, 20].

One of the concerns with graphically guided procedures is the cumulative radiation exposure for both medical personnel and patients, particularly in the case of repeated procedures [1]. Generally accepted figure is a 5% increase of risk of death from cancer with a 1 Sv (1000 mSv) dose [12]. According to the figure, the risk increase with the radiation exposure noted in our CT group would be 0.001%. The radiation exposure for the patients in the CT guided group was an order of magnitude lower than in some routine procedures – effective dose for a non-contrast head CT is approximately 2.1 mSv and 1.5 mSv for a single projection X-Ray of lumbar spine [14]. Low radiation exposure in our CT guided group (mean effective dose 0.21 mSv) can be attributed to the highly targeted short scans used in advanced conventional CT scanners. They provide high-precision visualization and require fewer slices, which reduces overall radiation exposure. Schmidt et al. described a conventional CT-guided procedure in 2020, anticipating a low radiation dose for the patient, although the actual dose was not reported [17]. Depending on scanning parameters, it is possible that their radiation doses were as low as those observed in our study.

General anesthesia is commonly used for this procedure; however, some patients are better suited for the percutaneous approach due to the higher risks associated with general anesthesia. At our institution, analgosedation was employed with no complications and was well tolerated by patients. The use of neuronavigation has been reported to extend surgery time by approximately 15–20 min, which could lead to discomfort for patients undergoing analgosedation [2].

We consider the learning curve for our approach to be very steep, based on several indicators: the procedure time, the number of CT scans needed to access the foramen ovale, and the pain relief outcomes remained consistent throughout the period we have been performing the procedure. Another indicator of the steep learning curve and simplicity of the method is that the fluoroscopy-guided procedures were conducted by a senior neurosurgeon, who was formerly the head of the department and had 30 years of experience. The CT-guided procedures were conducted by two younger neurosurgeons with 8 and 11 years of experience, respectively, with no prior training in percutaneous procedures. Nevertheless, they were able to achieve 100% success in cannulation of the foramen ovale with minimal complications in the CT-guided group.

By employing the CT-controlled approach described in this study, we minimize procedural risks, including the cumulative radiation dose for the patient, and the procedure under analgosedation is well tolerated. Therefore, in cases where pain relief is insufficient, the neurolysis can be repeated before considering alternative treatments, such as gamma knife irradiation.

We acknowledge, that the study may be limited by the small sample size and nonrandomized design.

## Conclusion

Low-dose CT-guided percutaneous rhizotomy conducted in the radiology suite carries negligible radiation exposure for patients and eliminates it for personnel. This method is fast, simple, precise, and carries a very low risk of complications.

## Data Availability

The data that support the findings of this study are available from the corresponding author upon reasonable request.
